# Energy metabolism in skeletal muscle cells from donors with different body mass index

**DOI:** 10.3389/fphys.2022.982842

**Published:** 2022-11-17

**Authors:** Parmeshwar B. Katare, Andrea Dalmao-Fernandez, Abel M. Mengeste, Håvard Hamarsland, Stian Ellefsen, Hege G. Bakke, Eili Tranheim Kase, G. Hege Thoresen, Arild C. Rustan

**Affiliations:** ^1^ Section for Pharmacology and Pharmaceutical Biosciences, Department of Pharmacy, University of Oslo, Blindern, Norway; ^2^ Faculty of Social and Health Sciences, Section for Health and Exercise Physiology, Inland Norway University of Applied Sciences, Lillehammer, Norway; ^3^ Department of Pharmacology, Institute of Clinical Medicine, University of Oslo, Oslo, Norway

**Keywords:** skeletal muscle cells, eicosapentaenoic acid, EPA, metabolism, obesity, mitochondria

## Abstract

Obesity and physical inactivity have a profound impact on skeletal muscle metabolism. In the present work, we have investigated differences in protein expression and energy metabolism in primary human skeletal muscle cells established from lean donors (BMI<25 kg/m^2^) and individuals with obesity (BMI>30 kg/m^2^). Furthermore, we have studied the effect of fatty acid pretreatment on energy metabolism in myotubes from these donor groups. Alterations in protein expression were investigated using proteomic analysis, and energy metabolism was studied using radiolabeled substrates. Gene Ontology enrichment analysis showed that glycolytic, apoptotic, and hypoxia pathways were upregulated, whereas the pentose phosphate pathway was downregulated in myotubes from donors with obesity compared to myotubes from lean donors. Moreover, fatty acid, glucose, and amino acid uptake were increased in myotubes from individuals with obesity. However, fatty acid oxidation was reduced, glucose oxidation was increased in myotubes from subjects with obesity compared to cells from lean. Pretreatment of myotubes with palmitic acid (PA) or eicosapentaenoic acid (EPA) for 24 h increased glucose oxidation and oleic acid uptake. EPA pretreatment increased the glucose and fatty acid uptake and reduced leucine fractional oxidation in myotubes from donors with obesity. In conclusion, these results suggest that myotubes from individuals with obesity showed increased fatty acid, glucose, and amino acid uptake compared to cells from lean donors. Furthermore, myotubes from individuals with obesity had reduced fatty acid oxidative capacity, increased glucose oxidation, and a higher glycolytic reserve capacity compared to cells from lean donors. Fatty acid pretreatment enhances glucose metabolism, and EPA reduces oleic acid and leucine fractional oxidation in myotubes from donor with obesity, suggesting increased metabolic flexibility after EPA treatment.

## 1 Introduction

Obesity and physical inactivity are important factors that predispose to type 2 diabetes and cardio-metabolic diseases (Kotsis et al., 2018). It has been reported that obesity leads to changes in the metabolic state of skeletal muscle cells including reduced insulin-mediated glucose uptake, oxidative metabolism, and lipid oxidative capacity [reviewed in (Wells et al., 2008; Tumova et al., 2016; Tallis et al., 2018)]. Given that skeletal muscle is the largest metabolic organ, any alteration in its energy utilization could have repercussions on the body’s energy homeostasis. Skeletal muscle constitutes approximately 40% of the body weight and contributes about 30% of resting energy expenditure (Frampton et al., 2020). It is a major organ for oxidation of fatty acids and carbohydrates (Erickson et al., 2021). At rest, 50% of energy consumption by skeletal muscle comes from fatty acids, while 28% comes from carbohydrates and the remaining 22% comes from amino acids (Melzer, 2011). However, under moderate exercise conditions, energy consumption from fatty acids increases up to 85% (Melzer, 2011). As fatty acids and carbohydrates are primary energy sources for skeletal muscle cells, changes in cellular energy metabolism may have a profound impact on overall energy generation and consumption by the body. These changes in the substrate metabolism in skeletal muscle cells during obesity could be attributed to mitochondrial dysfunction, changes in genes regulating insulin-mediated glucose uptake and lipid oxidation [reviewed in (Mengeste et al., 2021)].

We have previously reported that skeletal muscle cells from overweight individuals showed defects in insulin sensitivity and lipid handling as compared to cells from lean subjects (Feng et al., 2015; Løvsletten et al., 2020). Thus, the data revealed metabolic differences between skeletal muscle cells obtained from lean and subjects with overweight. Many metabolic processes are perturbed in skeletal muscle cells from individuals with obesity, and increased fatty acid accumulation and metabolites are thought to play a role in dysfunction of cellular insulin signaling and promoting insulin resistance (Eckardt et al., 2011; Mengeste et al., 2021). Studies looking into the metabolic differences of skeletal muscle cells between lean and donors with obesity are few. However, important findings are that myotubes from individuals with obesity have reduced or unaltered complete fatty acid oxidation compared to cells from lean individuals (Hulver et al., 2005; Holloway et al., 2007; Bell et al., 2010; Brault et al., 2010; Bucci et al., 2011). Basal glucose uptake and oxidation were increased in myotubes from overweight individuals compared to lean donors (Lund et al., 2017). In contrast, unchanged glucose oxidation in myotubes from individuals with obesity compared to lean donors has been reported (Gaster, 2007). This inconsistency might be attributed to differences in donor characteristics such as BMI. Furthermore, in the resting state, leg muscles in individuals with obesity had higher carbohydrate metabolism than those obtained from lean (Kelley et al., 1999). Christelle et al. reported a reduced protein turnover rate of skeletal muscle proteins and lack of stimulation of mitochondrial protein synthesis by insulin or amino acid in individuals with obesity (Guillet et al., 2009). Understanding these metabolic differences in intact skeletal muscle and cultured cells from lean and individuals with obesity and further elucidation of probable pathways is critical as these metabolic differences may contribute to the development of metabolic disorders and their progression (Petersen and Shulman, 2002).

It has been established that increased plasma free fatty acid concentration is associated with metabolic disorders and insulin resistance in humans (Xin et al., 2019). The amount and the type of fatty acids are of great importance for these perturbations. Diets rich in saturated fatty acids can cause metabolic imbalance and alterations in cellular signaling causing insulin resistance [reviewed in (Silva Figueiredo et al., 2017)]. However, intake of long-chain polyunsaturated n-3 fatty acids (n-3 PUFA) such as eicosapentaenoic acid (EPA) and docosahexaenoic acid (DHA) have been shown to improve lipid metabolism and prevent the development of obesity [reviewed in (Jeromson et al., 2015; Silva Figueiredo et al., 2017)]. We have previously observed that pretreatment of human skeletal muscle cells with EPA increased fatty acid and glucose uptake, and promoted triacylglycerol formation (Aas et al., 2006; Wensaas et al., 2009). The mechanism by which EPA affects skeletal muscle energy metabolism is not known in detail, but it may involve alterations in substrate metabolism. For instance, EPA has been suggested to increase futile substrate cycling in human myotubes, which could be of importance for the potential beneficial effects of n-3 PUFA on body weight regulation (Løvsletten et al., 2018).

As myotubes established from human biopsies are well differentiated and we have previously shown that they retain many phenotypic characteristics of the donors from which they have been derived (Aas et al., 2013; Løvsletten et al., 2018; Aas et al., 2020).They are well suited as an *in vitro* model system for studying the regulation of energy metabolism in skeletal muscle. In the present study, our primary focus was to study possible differences in glucose, fatty acid, and amino acid metabolism combined with proteomic analyses, as well as to evaluate responses to EPA treatment *in vitro*, in myotubes from lean and individuals with obesity.

## 2 Materials

Corning^®^ CellBIND^®^ tissue culture plates were from Corning (Schiphol-Rijk, the Netherlands). Dulbecco’s Modified Eagle’s Medium (DMEM) with GlutaMAX™ high and low glucose, Dulbecco’s Phosphate Buffered Saline (DPBS; with Ca^2+^ and Mg^2+^), heat-inactivated foetal bovine serum (FBS), penicillin-streptomycin (10,000 IE/ml), amphotericin B, human epidermal growth factor (hEGF), trypsin-EDTA, Restore™ PLUS Western Blot stripping buffer, Super Signal™ West Femto Maximum Sensitivity substrate, Pierce™ BCA Protein Assay Kit, Power SYBR^®^ Green PCR Master Mix, TaqMan reverse transcription kit reagents, High-Capacity cDNA Reverse Transcription Kit, MicroAmp^®^ Optical 96-well Reaction Plate, MicroAmp^®^ Optical Adhesive Film, and primers for TaqMan PCR were purchased from Thermo Fisher Scientific (Waltham, MA, United States). Insulin (Actrapid^®^ Penfill^®^ 100IE/ml) was from NovoNordisk (Bagsvaerd, Denmark). D-[^14^C(U)]glucose (3.0 mCi/mmol), [1–^14^C]oleic acid (OA, 59.0 mCi/mmol) and L-[^14^C(U)]leucine (59.0 mCi/mmol) were from PerkinElmer NEN^®^ (Boston, MA, United States). Ultima Gold™ XR, Pico Prias 6 ml PE vials, 96-well Isoplate^®^, UniFilter^®^-96 GF/B microplates, and TopSeal^®^-A transparent film were obtained from PerkinElmer (Shelton, CT, United States). 4-(2-hydroxyethyl)-1-piperazineethanesulfonic acid (HEPES), β-mercaptoethanol, dimethyl sulfoxide (DMSO), bovine serum albumin (BSA), dexamethasone, gentamicin, l-glutamine, l-carnitine, protease inhibitor, phosphatase II inhibitor, trypan blue 0.4% solution d-glucose, oleic acid (OA, 18:1, n-9), eicosapentaenoic acid (EPA, 20:5, n-3) and palmitic acid (16:0) were obtained from Sigma-Aldrich (St. Louis, MO, United States). QIAshredder and RNeasy Mini Kit were from QIAGEN (Venlo, the Netherlands).

## 3 Ethics statement

The studies involving human participants were reviewed and approved by Regional Committee for Medical and Health Research Ethics: reference number REK11959. The patients/participants provided their written informed consent to participate in this study.

## 4 Methods

### 4.1 Donor characteristics

Cultured myotubes used were established from biopsies from 24 adult donors, both females and males, age 47 ± 3 years 14 lean donors (body mass index (BMI) < 25 kg/m^2^) and ten donors with obesity (BMI >30 kg/m^2^) were included. A summary of selected donor characteristics is given in Table 1 below.

**TABLE 1:** Donor characteristics. Results present mean ± SEM. Statistical significance was calculated as the difference between for two groups, and these differences were tested by paired *t* test. *p < 0.05 vs. lean donors BMI, body mass index, F, female; M, male.

Donor characheristics				
Group	Sample size	Age	BMI	Gender
Lean donors	14	48 ± 3	24 ± 0.5	F-8, M-6
Donors with obesity	10	48 ± 0.4	35 ± 1*	F-5, M-5

### 4.2 Cell culture

Human satellite cells were isolated from muscle biopsy samples from *musculus vastus lateralis* from lean donors and donors with obesity as previously described (Lund et al., 2018; Hammarström et al., 2020; Mølmen et al., 2021). In brief, satellite cells were isolated from muscle biopsies, decontaminated of fibroblasts and grown to three to six passages. The isolated cells were cultured and proliferated in DMEM-GlutaMAX (5.5 mM glucose) supplemented with 10% FBS, HEPES (25 mM), gentamicin (50 ng/ml), penicillin (25 IU), streptomycin (25 μg/ml), amphotericin B (1.25 μg/ml), hEGF (10 ng/ml), dexamethasone (0.39 μg/ml) and 0.05% BSA. Differentiation of myoblasts into myotubes was induced at 80–90% confluence by changing the medium to DMEM-GlutaMAX (5.5 mM glucose) supplemented with 2% FBS and 25 p.m. insulin. Proteomic analysis confirmed that cells from all donors expressed markers of myotubes. The protein expressions of these markers were not statistically different in myotubes from lean donors and donors with obesity. This indicated that the myotubes from both groups were well differentiated and exhibited the basic characteristics of skeletal muscle cells. The cells were cultured at 37°C in a humidified atmosphere containing 5% CO_2_, and the medium was changed regularly every 2–3 days. On sixth day, cells were treated with 100 μM of either eicosapentaenoic acid (EPA) or palmitic acid (PA) complexed to fatty acid-free BSA at ratio 2.5/1. Experiments were carried out 7–8 days after the induction of cell differentiation.

### 4.3 Substrate oxidation assay

Skeletal muscle cells were cultured in 96-well CellBIND^®^ microplates. The cells were then given D-[^14^C(U)]glucose (0.5 μCi/ml, 200 μM) or [1^−14^C]oleic acid (0.5 μCi/ml, 100 μM) or L-[^14^C(U)]leucine (0.5 μCi/ml, 800 μM) substrate during 4 h CO_2_-trapping as described previously (Wensaas et al., 2007). The glucose substrate was prepared in DPBS supplemented with HEPES (10 mM) and BSA (10 μM), whereas the oleic acid substrate was added in DPBS containing HEPES (10 mM), BSA (40 µM), and l-carnitine (1 mM). Following trapping, the ^14^CO_2_ produced by the cells and cell-associated (CA) radioactivity were measured using a 2450 MicroBeta2 liquid scintillation counter (PerkinElmer). Protein concentration in each well was determined with the Bio-Rad protein assay kit to relate the ^14^CO_2_ and CA data to cellular protein content. Complete substrate oxidation was measured as ^14^CO_2_ and uptake was calculated by ^14^CO_2_ + CA. The ratio between ^14^CO_2_ and uptake was identified as fractional substrate oxidation (^14^CO_2_/uptake).

### 4.4 Proteomic analysis

Human skeletal muscle cells were cultured and differentiated in 25 cm^2^ flasks. On day 7 of the differentiation period, the cells were washed with DPBS containing Mg^2+^ and Ca^2+^ before harvested, washed in DPBS, and spun down at 1,000 rpm at 4°C for 5 min. The cells were then snap frozen in liquid nitrogen and stored at −80°C. The cells were lysed using 150 μL of RIPA buffer containing protease inhibitor followed by protein aggregation, and protein reduction, alkylation, and digestion into peptides with trypsin. Resulting peptides were desalted and concentrated before mass spectrometry by the STAGE-TIP method using a 3M Empore™ C18 resin disc. LC-MS/MS analysis was carried out using a nanoElute nanoflow ultrahigh pressure LC system (Bruker Daltonics, Bremen, Germany) coupled to the timsTOF fleX mass spectrometer (Bruker Daltonics), using a CaptiveSpray nanoelectrospray ion source (Bruker Daltonics). 200 ng of peptide digest was loaded on a capillary C18 column (25 cm length, 75 μm inner diameter, 1.6 μm particle size, 120 Å pore size; IonOpticks, Fitzroy, VIC, Australia). Resulting MS raw files were submitted to the MaxQuant software version 2.0.1.0 for protein identification and label-free quantification. Carbamidomethyl (C) was set as a fixed modification and acetyl (protein N-term), carbamyl (N-term), and oxidation (M) were set as variable modifications. First search peptide tolerance of 20 ppm and main search error of 6 ppm were used. Trypsin without proline restriction enzyme option was used, with two allowed miscleavages. The minimal unique þ razor peptides number was set to 1, and the allowed FDR was 0.01 (1%) for peptide and protein identification. Label-free quantitation was employed with default settings. UniProt database with ‘human’ entries (September 2021) was used for the database searches. Known contaminants as provided by MaxQuant and identified in samples were excluded from further analysis. Gene Ontology enrichment analysis for biological processes was performed using STRING software (https://string-db.org/). The mass spectrometry proteomics data have been deposited to the ProteomeXchange Consortium via the PRIDE [1] partner repository with the dataset identifier PXD037909.

## 5 Statistical analysis

All values are presented as mean ± SEM unless stated otherwise in the figure legends. The value *n* represents the number of individual experiments with four to eight wells for each condition in each experiment. Statistical analysis and graphs were performed using GraphPad Prism 8.0.1 for Windows (GraphPad Software Inc. San Diego, CA, United States). The differences between the groups were compared using either *t* test or ANOVA followed by Tukey test and mentioned in the respective figure legends. *p* ≤ 0.05 was considered significant.

## 6 Results

### 6.1 Proteomic analysis

**FIGURE 1:**
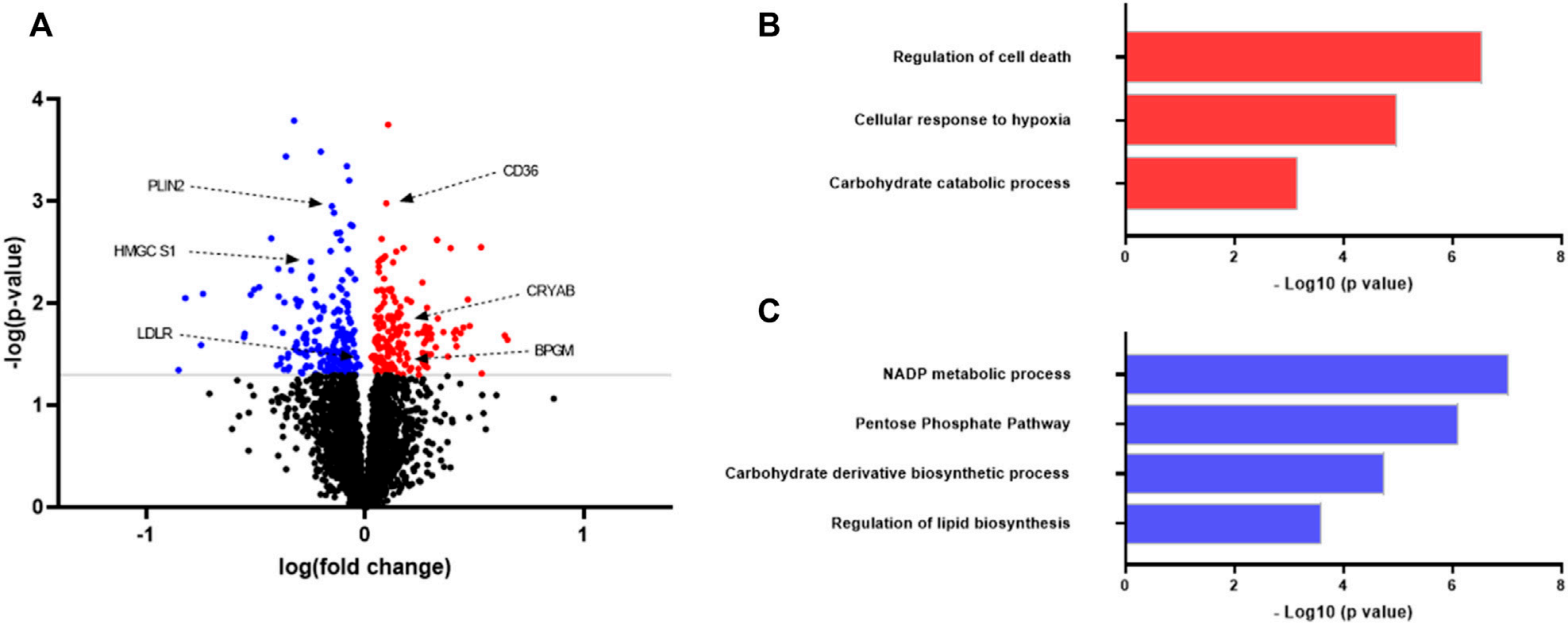
Proteomic analysis of protein expression in myotubes from lean donors and donors with obesity. Human myoblasts were grown and differentiated to myotubes in 25 cm^2^ cell culture flasks. On day 7 of differentiation the myotubes from eight different donors in each group were harvested for proteomic analysis. Lean group was considered as a control for the comparison. **(A)** Volcano plot of differentially regulated proteins. Some proteins mentioned in the text are shown. Statistical significance was calculated as the difference between the two groups using unpaired *t* test, and these differences were considered significant if *p* < 0.05. **(B)** Upregulated biological processes in myotubes from individuals with obesity as compared to lean. **(C)** Downregulated biological processes in myotubes from individuals with obesity as compared to lean. Results from gene ontology (biological processes) analysis performed using STRING software (https://string-db.org/). Selected differently regulated pathways related to metabolism and signaling. CD36, cluster of differentiation 36; PLIN2, perilipin two; BPGM, biphosphoglycerate mutase; LDLR, low-density lipoprotein receptor; HMGCS1, hydroxymethylglutaryl-CoA synthase; CRYAB, alpha-crystallin B chain.

To study possible differences between myotubes established from lean donors and donors with obesity, proteomic analysis of cell lysates was performed (Figure 1; Supplementary Table S1, S2). The proteome of myotubes was investigated using quantitative label-free proteomics. The analysis detected more than 3700 proteins, of which 172 were significantly upregulated (Supplementary Table S1) and 199 were significantly downregulated (Supplementary Table S2) in myotubes from donors with obesity compared to myotubes from lean donors (Figure 1A).

With the focus on elucidating how the signaling pathways were altered in myotubes, Gene Ontology enrichment of regulated proteins was performed. Myotubes from donors with obesity showed increased expression of proteins regulating the hypoxia response compared to cells from lean donors (Figure 1B). Corroborating with this, myotubes from subjects with obesity showed several proteins regulating the cellular stress response to be upregulated, including mitogen-activated protein (MAP) kinase 1, sulfiredoxin-1 and glutathione peroxidase one indicating increased cellular oxidative stress in myotubes from this donor group (Supplementary Table S1). Several enzymes involved in glycolysis pathway (carbohydrate catabolic processs), such as phosphoglycerate kinase one and biphosphoglycerate mutase (BPGM) were upregulated in cells from subjects with obesity (Supplementary Table S1), whereas the pentose phosphate pathway (PP pathway) was downregulated (Figure 1C). Protein expression of an important rate-limiting enzyme from the PP pathway glucose-6-phosphate dehydrogenase (G6PD) was observed to be downregulated. Apart from this, protein expression of enzymes involved in the glycogenolysis process was found to be upregulated in myotubes from subjects with obesity (Supplementary Table S1).

Myotubes from donors with obesity showed increased expression of several proteins from apoptosis pathway, including tumor protein p53-inducible protein three and yes1 associated transcriptional regulator (Figures 1A,B; Supplementary Table S1). Total 34 proteins involved in cellular apoptotic pathway were found to be upregulated in myotubes from donors with obesity as compared to cells from lean donors (Supplementary Table S2).

Key regulator proteins from lipid biosynthetic pathway, including hydroxymethylglutaryl-CoA synthase (HMGCS1) and low-density lipoprotein receptor (LDLR) were found to be downregulated in myotubes from donors with obesity compared to cells from lean donors (Figure 1; Supplementary Table S2). Cluster of differentiation 36 (CD36) was significantly upregulated in myotubes from donors with obesity. Furthermore, long-chain fatty acid transport protein 3 (SLC27A3) and perilipin 2 (PLIN 2) were downregulated in myotubes from this group of donors.

### 6.2 Fuel handling experiments

**FIGURE 2:**
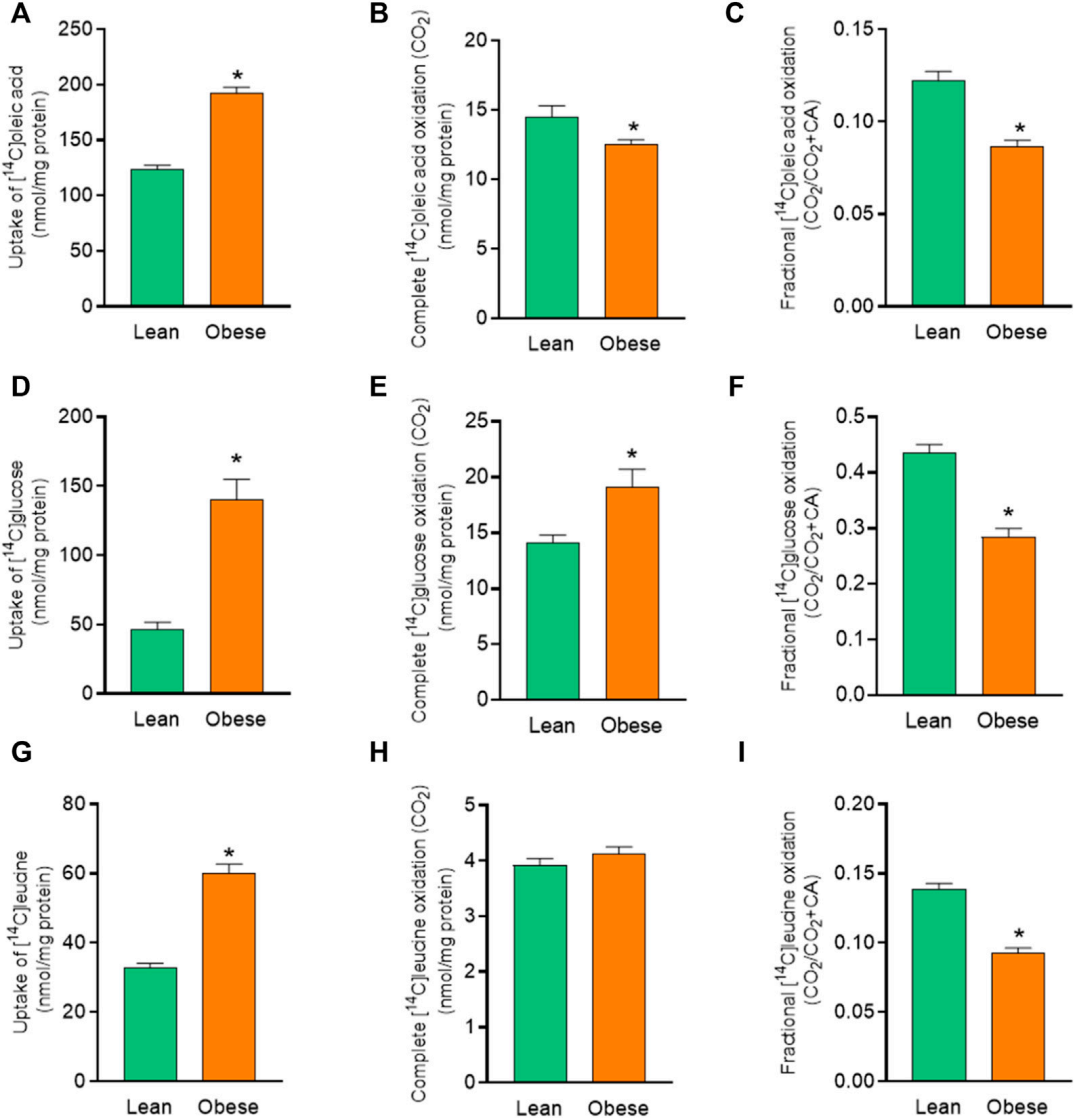
Overall substrate metabolism in myotubes established from lean and donors with obesity. Differentiated skeletal muscle cells (myotubes) from lean or donors with obesity were incubated with [^14^C]oleic acid (100 µM), [^14^C]glucose (200 µM) and [^14^C]leucine (800 µM) for 4 h. The figures show: **(A)** total uptake of OA by the cells (CO_2_+CA), **(B)** complete oxidation of OA to carbon dioxide (CO_2_), **(C)** fractional OA oxidation (CO_2_/uptake), **(D)** total uptake of glucose by the cells (CO_2_ +CA), **(E)** complete oxidation of glucose to carbon dioxide (CO_2_), **(F)** fractional glucose oxidation (CO_2_/uptake), **(G)** total uptake of leucine by the cells (CO_2_+CA), **(H)** complete oxidation of leucine to carbon dioxide (CO_2_), **(I)** fractional leucine oxidation ((CO_2_/uptake). The results shown represent the combined substrate metabolism for all treatments (overall effect) in myotubes from lean and donors with obesity. Results present means ± SEM (*n* = 12). Statistical significance was calculated as the difference between the two groups using unpaired *t* test. *p ≤ 0.05 vs. myotubes from lean donors.

To further elucidate the differences in energy substrate utilization between muscle cells from the two donor groups, uptake and oxidation of radiolabeled oleic acid (OA), glucose, and leucine were studied. Myotubes from donors with obesity showed an increased OA uptake and a reduced OA oxidation compared to myotubes from lean donors (Figures 2A,B). Fractional oxidation of OA was markedly reduced in cells from donors with obesity (Figure 2C) indicating that the increased uptake of OA was not met by increased oxidation, suggesting enhanced cellular accumulation of OA.

The effect of obesity was more profound on glucose uptake. There was 3-fold increased glucose uptake in myotubes from donors with obesity (Figure 2D) compared to cells from lean donors. Myotubes from donors with obesity also showed an overall increase in glucose oxidation compared to cells from lean donors (Figure 2E). Fractional oxidation of glucose on the other hand was reduced in myotubes from donors with obesity compared to cells from lean donors (Figure 2F). This showed that the increased uptake of glucose was higher than the increased oxidation, leading to an increased level of cell-associated glucose.

There was also an increase in leucine uptake in myotubes from donors with obesity compared to cells from lean donors (Figure 2G). However, there was no significant difference in leucine oxidation between myotubes from the two donor groups (Figure 2H). Furthermore, myotubes from donors with obesity accumulated more leucine as indicated by reduced fractional leucine oxidation (Figure 2I).

### 6.3 Effects of pretreatment with fatty acids

**FIGURE 3:**
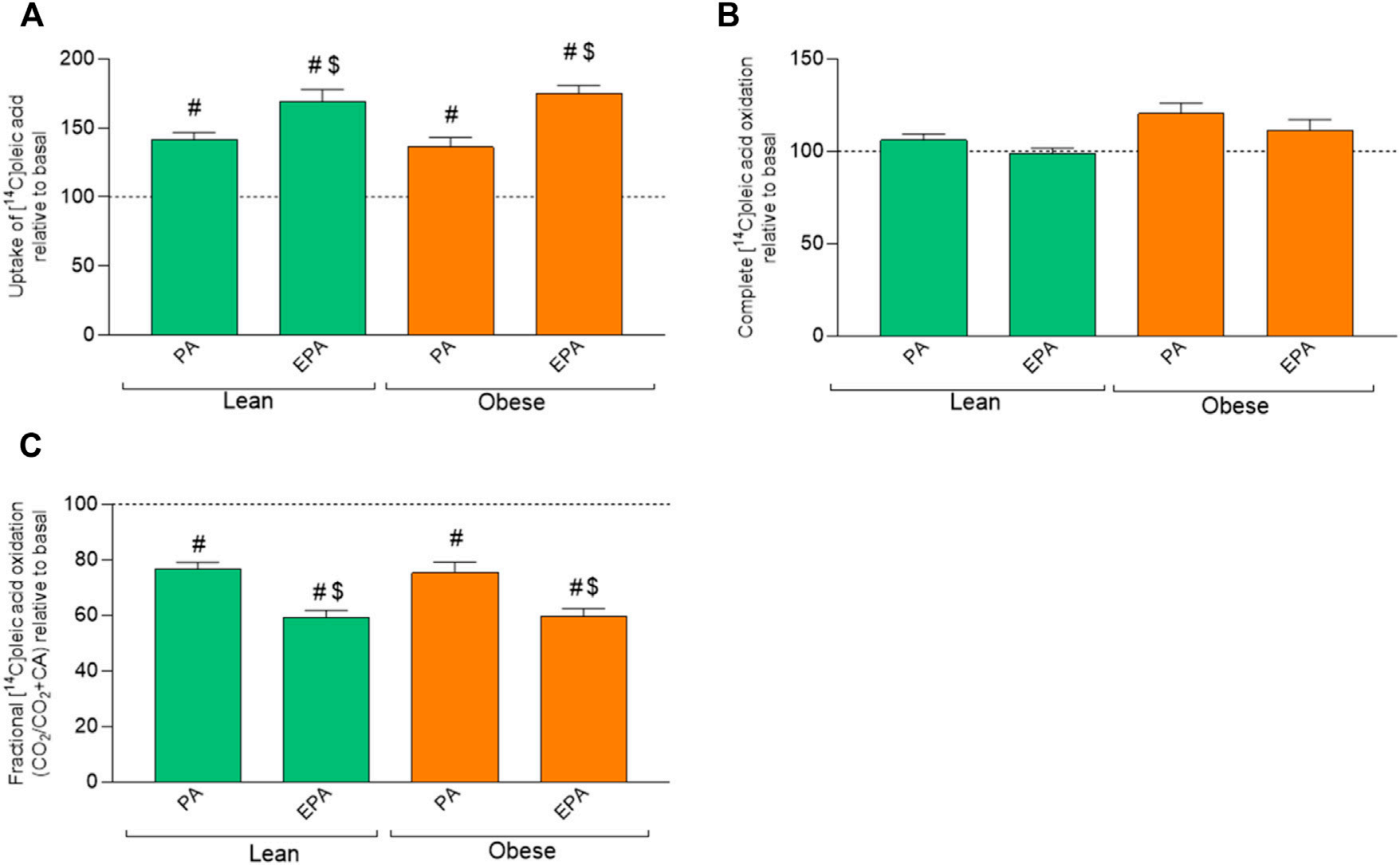
Effects of preincubation with fatty acids on oleic acid uptake and oxidation. Differentiated skeletal muscle cells (myotubes) from lean or donors with obesity were preincubated with either no fatty acid (basal), PA (100 µM) or EPA (100 µM) for 24 h, and then incubated with [1^−14^C]oleic acid (100 µM) for 4 h. The figures show: **(A)** total uptake of OA in the cells (CO_2_+CA), **(B)** complete oxidation of OA to carbon dioxide (CO_2_), **(C)** fractional OA oxidation (CO_2_/uptake). Results present mean ± SEM (*n* = 8). Results for lean group, basal (without treatment) was set to 100%, and for obese group, basal (without treatment) was set to 100%. Statistical significance was calculated as the difference between the two groups using one way ANOVA. ^#^ *p* ≤ 0.05 vs. basal for myotubes from the same group, ^$^ *p* ≤ 0.05 vs. respective PA in cells from the same group.

To study whether a challenge with fatty acids would modify energy metabolism, 100 µM of palmitic acid (PA) or 100 µM eicosapentaenoic acid (EPA) was added during the last 24 h of cell culturing. Pretreatment with EPA and PA increased OA uptake in myotubes from both donors groups compared to basal (no fatty acid pretreatment) (Figure 3A), but did not show any effect on OA oxidation (Figure 3B). Furthermore, OA uptake was significantly higher after EPA treatment also when compared to PA (Figure 3A). PA and EPA both reduced fractional oxidation in myotubes from both lean donors and donors with obesity compared to basal (Figure 3C). Furthermore, OA fractional oxidation was significantly reduced after EPA treatment also when compared to PA (Figure 3C).

**FIGURE 4:**
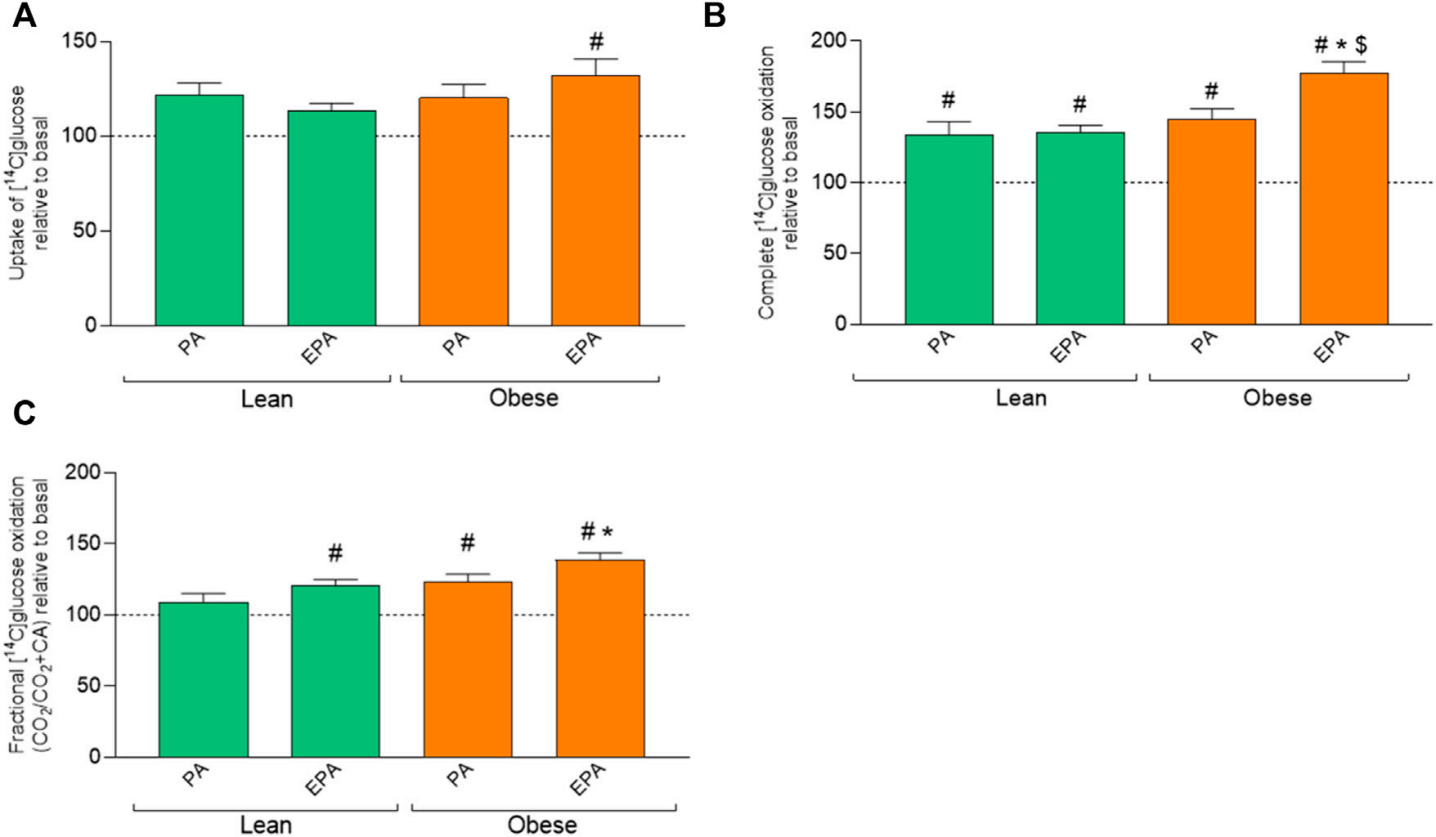
Effects of pretreatment with fatty acids on glucose metabolism in myotubes from lean and donors with obesity. Differentiated skeletal muscle cells (myotubes) from lean or donors with obesity were pretreated with either no fatty acid (basal), PA (100 µM) or EPA (100 µM) for 24 h, and then incubated with [^14^C]glucose (200 µM) for 4 h. The figures show: **(A)** total uptake of glucose by the cells (CO_2_+CA), **(B)** complete oxidation of glucose to carbon dioxide (CO_2_), **(C)** fractional glucose oxidation (CO_2_/uptake). Results present means ± SEM (*n* = 8). Results for lean group, basal (without treatment) was set to 100%, and for obese group, basal (without treatment) was set to 100%. Statistical significance was calculated as the difference between the two groups using one way ANOVA, and these differences were considered significant if ^#^ *p* ≤ 0.05 vs. basal from same group, ^*^ *p* ≤ 0.05 vs. respective treatment for lean group and ^$^ *p* ≤ 0.05 vs. PA from the same group.

Only EPA pretreatment significantly increased glucose uptake in myotubes from donors with obesity compared to basal (Figure 4A). EPA and PA increased glucose oxidation in muscle cells from both donor groups (Figure 4B). Furthermore, glucose oxidation was significantly higher after EPA treatment also when compared to PA in myotubes from donors with obesity (Figure 4B). EPA in lean and both PA and EPA in the obese group increased glucose fractional oxidation compared to basal (Figure 4C). Furthermore, glucose fractional oxidation was significantly increased after EPA treatment also when compared to PA in myotubes from donors with obesity (Figure 4C).

**FIGURE 5:**
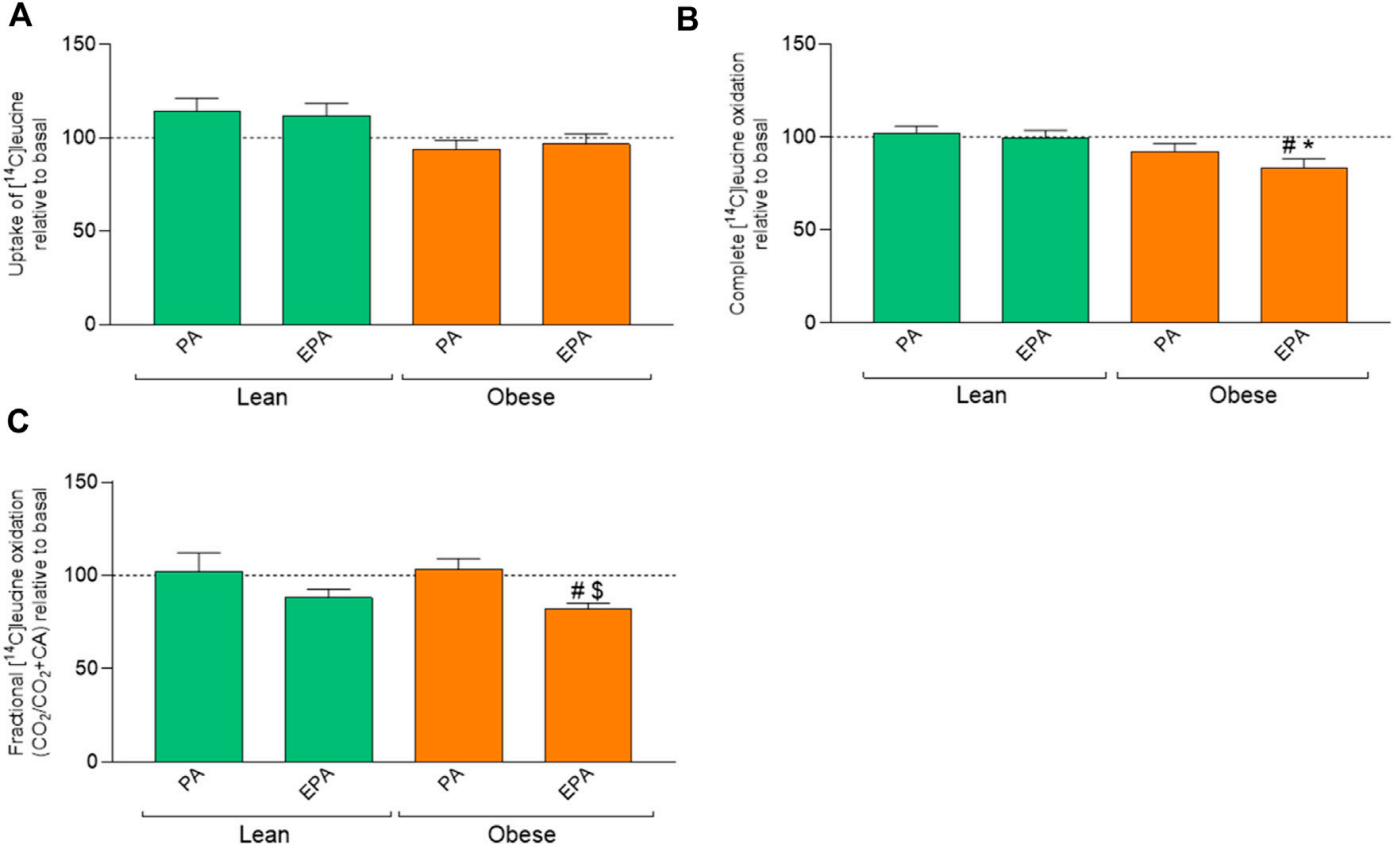
Effects of preincubation with fatty acids on leucine metabolism in myotubes from lean and donors with obesity. Differentiated skeletal muscle cells (myotubes) from lean or donors with obesity were preincubated with either no fatty acid (basal), PA (100 µM) or EPA (100 µM) for 24 h, and thereafter incubated with [^14^C]leucine (800 µM) for 4 h. The figures show: **(A)** total uptake of leucine in the cells (CO_2_+CA), **(B)** complete oxidation of leucine to carbon dioxide (CO_2_), and **(C)** fractional leucine oxidation (CO_2_/uptake). Results present means ± SEM (*n* = 8). Results for lean group, basal (without treatment) was set to 100%, and for obese group, basal (without treatment) was set to 100%. Statistical significance was calculated as the difference between the two groups using one way ANOVA, and these differences were considered significant if ^#^ *p* ≤ 0.05 vs. basal for cells from the same group, ^*^ *p* ≤ 0.05 vs. respective treatment for the lean group and ^$^ *p* ≤ 0.05 vs. PA from the same group.

Pretreatment with fatty acids did not induce any effect on leucine uptake (Figure 5A). However, pretreatment of myotubes from donors with obesity with EPA showed lower oxidation of leucine compared to basal as well as EPA treatment of cells from the lean group (Figures 5B,C) indicating an increased relative accumulation of leucine after EPA pretreatment.

## 7 Discussion

In the present study, we showed by proteomic analysis of cell lysate that carbohydrate catabolic process, cellular response to hypoxia, and pathway regulating cell death were upregulated in myotubes from donors with obesity compared to cells from lean donors. Key regulatory proteins of glucose metabolism such as phosphoglycerate kinase one and biphosphoglycerate mutase (BPGM) were upregulated, while the pentose phosphate pathway and lipid biosynthetic pathway were downregulated in muscle cells from obese donors. In addition, substrate uptake of OA glucose and leucine was increased in myotubes from donors with obesity. OA oxidation was reduced, while glucose uptake and oxidation were increased and oxidation of leucine was unchanged in myotubes from donors with obesity compared to cells from lean donors. Pretreatment with both PA and EPA increased OA accumulation in cells from donors with obesity, increased glucose oxidation, and decreased OA fractional oxidation in cells from both donor groups. While only EPA pretreatment increased glucose uptake and decreased oxidation of leucine in myotubes from donors with obesity. These results from the experiments in the study could help us understand more about the substrate preferences and metabolic capacity of skeletal muscle cells obtained from individuals with obesity.

Proteomic data showed that many important proteins in the lipid biosynthetic pathway were downregulated in myotubes from donors with obesity including hydroxymethylglutaryl-CoA (HMG-CoA) synthase, 3-beta-hydroxysteroid-delta (Mengeste et al., 2021) isomerase (EBP) and corticosteroid 11-beta-dehydrogenase isozyme 1 (HSD11B1). The low density lipoprotein (LDL) receptor, which is responsible for the uptake of cholesterol-carrying lipoproteins (Jeon and Blacklow, 2005), was also downregulated. Downregulation of lipid biosynthetic pathway and LDL receptor could be a response in skeletal muscle cells due to higher plasma fatty acid levels due to obesity (Fernandez and West, 2005). Reduced LDL receptor activity is correlated with an increase in LDL cholesterol (Applebaum-Bowden et al., 1984). Furthermore, cluster of differentiation 36 (CD36) receptor responsible for cellular uptake of long-chain fatty acids was upregulated. Studies have previously reported an increased expression of CD36 in adipose tissue during obesity (Bonen et al., 2006) and in individuals with high intrahepatic triacylglycerol levels (Fabbrini et al., 2009). Lipid accumulation in obesity and diabetes is also associated with increased CD36 expression in skeletal muscle (Bonen et al., 2004; Aguer et al., 2010). Both perilipin 2 (PLIN2) and long-chain fatty acid transport protein 3 (SLC27A3) were found to be downregulated in myotubes from donors with obesity. Studies have suggested that downregulation of PLIN2 may alter lipid metabolism and promote cellular accumulation of lipids (Conte et al., 2019; Mardani et al., 2019). A preclinical study in mice showed that long-chain fatty acid transport protein 3 (SLC27A3) knockdown decreased fatty acid activation but not uptake. The reduced activation of fatty acids could be attributed to the reduced acyl-CoA synthetase activity in this model (Pei et al., 2004).

To explore how the proteomic changes in lipid metabolic pathways affected fatty acid metabolism, we looked into OA uptake and oxidation in myotubes. It has previously been reported that myotubes established from lean donors and donors with obesity retain donor characteristics *in vitro* related to lipid metabolism (Gaster et al., 2002; Gaster et al., 2004; Aas et al., 2013; Mengeste et al., 2021). Myotubes from individuals with obesity oxidized less and accumulated more OA than cells from lean donors. Previous studies have observed differences in lipid metabolism in myotubes from donors with obesity (Kelley et al., 1999). Myotubes from donors with obesity showed higher fatty acid uptake (Løvsletten et al., 2020), which could be attributed to greater protein facilitated apparatus for fatty acid transport (Bonen et al., 2004). We found a 3-fold increased expression of CD36 in myotubes from donors with obesity in the present study. Reduced fatty acid oxidation has been reported in intact muscle strips and muscle homogenates of individuals with severe obesity (Kim et al., 2000; Houmard et al., 2012). Furthermore, lipid oxidation efficiency was found to be reduced in skeletal muscle cells harvested from donors with obesity (Bell et al., 2010). Furthermore, reduced fatty acid oxidation efficiency was found to be correlated with *in vivo* measurements in skeletal muscle (Bell et al., 2010). The reduced fatty acid oxidation in myotubes from individuals with obesity could be due to reduced mitochondrial content, as indicated by reduced mtDNA copy number (Houmard et al., 2012). However, fatty acid oxidation was equivalent in muscle cells from lean and individuals with severe obesity after correcting for mtDNA copy number (Consitt et al., 2010). These data indicate that individuals with obesity have normally functioning mitochondria and that the reduced fatty acid oxidation capacity could be caused by reduced mitochondrial content. Moreover, reduced fatty acid oxidation in skeletal muscle in individuals with severe obesity has been attributed to impairments at multiple stages of fatty acid oxidation, including impaired fatty acid transport, reduced mitochondrial content, and reduced efficiency of fatty acid beta oxidation (Houmard et al., 2011; Boyle et al., 2012; Houmard et al., 2012; Fritzen et al., 2020). EPA and PA treatment of myotubes from both donors increased OA uptake compared to a fatty acid-free control (basal). These findings are consistent with previous studies by us, where we observed that preincubation with EPA promoted increased uptake of both OA and PA in myotubes from obese donors with diabetes as well as in cells from lean subjects (Wensaas et al., 2009; Løvsletten et al., 2018). EPA significantly increased OA accumulation in myotubes from both donor groups as compared to basal and PA. EPA has been shown to increase FA accumulation and turnover in human myotubes to increase futile substrate cycling, which could be of importance for the potential beneficial effects of n-3 PUFA on body weight regulation (Løvsletten et al., 2018). Moreover, reduced fractional oxidation after EPA and PA treatment indicated increased accumulation of OA in myotubes from both donor groups. The proteomic analysis further shed light on changes in the expression of important enzymes involved in metabolic pathways. These perturbations in the metabolic pathways in myotubes are important manifestations of rising BMI and could be indispensable to cope with the changing substrate availability.

Proteomic data suggested that protein expression of important proteins and enzymes involved in carbohydrate metabolic pathways were perturbed in myotubes from donors with obesity. The pentose phosphate pathway and carbohydrate derivative biosynthetic process were downregulated. Nonetheless, carbohydrate catabolic processes including glycolysis and glycogenolysis were upregulated. These findings suggested that myotubes from donors with obesity have perturbed carbohydrate metabolism. Hittel et al. (2005) have reported increased protein expression of glycolytic enzymes in females with obesity, suggesting compensatory glycolytic drift to counteract reduced fatty acid oxidation. Similarly, we observed increased expression of enzymes from glycolytic pathway including biphosphoglycerate mutase and phosphoglycerate kinase 1, indicating similar glycolytic drift in myotubes from donors with obesity. Furthermore, it has been reported that lactate generation is increased in muscle strips under resting state in donors with obesity (Friedman et al., 1994). Alpha-crystallin B chain, an important protein in response to a decreased oxygen level in the cells was up 2-fold-increased in myotubes from donors with obesity indicating a possible cellular hypoxic condition. The upregulated hypoxic pathway in these myotubes indicates adaption to lower oxygen levels. Reduced oxygen content in the cells could lead to reduced efficiency of mitochondrial respiration, making cells more reliant on glycolysis for ATP generation. Furthermore, downregulation of pentose phosphate pathway could be an adaptation of the myotubes to divert more glucose and metabolites from the glycolysis pathway to increase ATP generation under hypoxic conditions. However, this may reduce NADPH in the cell and disturb redox balance, as indicated by increased expression of several proteins regulating the cellular stress, including mitogen-activated protein (MAP) kinase 1, sulfiredoxin-1, and glutathione peroxidase one in myotubes from donors with obesity. A previous study with a high-fat diet-induced obesity mice model has shown reduced activity of the pentose phosphate pathway (Stucchi et al., 2012). The overall perturbations and adaptations in carbohydrate metabolic pathways give myotubes from donors with obesity an increased ability to process glucose for ATP generation even under hypoxic conditions.

Bourlier et al. (2013) reported increased glucose metabolism in skeletal muscle cells from donors with obesity after 8 weeks of exercise intervention. Our previous research has shown that myotubes from overweight donors had increased glucose uptake and oxidation as compared to normal weight donors (Lund et al., 2017). However, several previous studies showed no significant donor-related differences in basal glucose oxidation in myotubes (Kase et al., 2005; Kase et al., 2007; Feng et al., 2015). In the present study, we observed increased glucose uptake and oxidation in myotubes from donors with obesity. Fractional oxidation of glucose was reduced in cells from donors with obesity indicating enhanced accumulation of glucose in these cells. We have previously reported that treatment of skeletal muscle cells with EPA upregulated the pentose phosphate pathway (Hessvik et al., 2010), indicating EPA treatment could increase the metabolic flexibility of skeletal muscle cells. Metabolic flexibility is diminished by obesity (Blaak et al., 2000; Corpeleijn et al., 2008). EPA can improve the adaptability of myotubes to changing substrate availability (Hessvik et al., 2010). Fatty acid pretreatment increased glucose oxidation in myotubes from both donor groups. Consistent with our results, it has previously been reported that EPA treatment of human myotubes from healthy individuals increased glucose and fatty acid uptake (Aas et al., 2006; Løvsletten et al., 2018).

Not much research has been done to understand the effect of obesity on amino acid uptake, protein synthesis, and turnover in human skeletal muscle cells. Recent research suggests that protein synthesis could be altered in skeletal muscle in individuals with obesity (Beals et al., 2019; Freitas and Katsanos, 2022). However, there is a large heterogeneity when it comes to protein metabolism in skeletal muscle from subjects with obesity. In the present study, leucine oxidation was not different in myotubes between lean and donors with obesity. This indicated that obesity did not affect amino acid oxidation. However, leucine uptake was higher and fractional oxidation was reduced in myotubes from donors with obesity, suggesting that these cells accumulated more leucine than cells from lean subjects. Moreover, EPA treatment promoted reduced fractional oxidation of leucine in myotubes from individuals with obesity, suggesting that EPA treatment may promote amino acid accumulation and thereby protein synthesis in myotubes. Previously, Smith et al. (2004) reported that EPA treatment of cachectic mice reduced skeletal muscle protein degradation by 88% but did not have any effect on protein synthesis. Furthermore, EPA may decrease protein breakdown by decreasing the expression of proteasome subunits (Murphy et al., 2011). The mechanisms behind the donor group and fatty acid effects on protein metabolism in human skeletal muscle cells have to be further elucidated.

Apart from metabolic pathways, apoptotic pathway was highly upregulated in myotubes from donors with obesity. A total of 34 proteins involved in cellular apoptotic pathways were found to be upregulated in cells from donors with obesity as compared to myotubes from lean. This could indicate increased skeletal muscle apoptosis in cells from donors with obesity. Studies have reported increased apoptosis of skeletal muscle cells in subjects with obesity (Akhmedov and Berdeaux, 2013; Heo et al., 2018). There has also been reported increased apoptosis in skeletal muscle in a diet-induced model of obesity in rats (Sishi et al., 2011). The increased oxidative stress during obesity could be the primary reason behind a possibly increased apoptosis of skeletal muscle cells (Heo et al., 2018).

The study can help us understand how higher BMI can affect metabolic capability and flexibility of skeletal muscle cells. EPA treatment of myotubes from individuals with obesity increased OA and glucose uptake and glucose oxidation indicating EPA may modify metabolic flexibility in these cells.

The advantages of using myotubes include consistency of results and easier manipulations *in vitro*. Performing the mechanistic study to elucidate the cellular mechanism becomes much easier using myotube. Although our results showed metabolic and proteomic differences between myotubes from lean donors and donors with obesity, there are some limitations of this study, which should be considered. Mature human myotubes are quiescent in culture and do not contract unless stimulated (Marš et al., 2021). However, myotubes are not immortalized and retains innate donor characteristics (Aas et al., 2013), thereby offering the possibility of studying innate characteristics of the donor including insulin resistance, glucose uptake, and substrate oxidation. Another limitation of the present study is that, we used a single dose of the fatty acids for the treatment in the myotubes. Using increasing concentration of fatty acids could have assisted to explain the dose dependent effect on the myotubes. Thus paving the way for future research possibilities in this direction.

## 8 Conclusion

Our study showed that myotubes from donors with obesity showed increased fatty acid, glucose and amino acid uptake when compared to cells from lean donors. Myotubes from donors with obesity had reduced fatty acid oxidative capacity, increased glucose oxidation and a higher glycolytic reserve capacity. This adaptation in carbohydrate metabolism may be important to mitigate the effect of hypoxia and reduced fatty acid oxidation on energy metabolism and ATP generation in skeletal muscle from donors with obesity (Stapleton et al., 2008). EPA pretreatment increased OA uptake in both donors and glucose oxidation and uptake in donors with obesity, indicating EPA can modify metabolism of glucose and OA in myoutubes. However, EPA reduced leucine oxidation in myotubes from donors with obesity, suggesting that an effect of EPA may be to promote amino acid conservation.

## Data Availability

The datasets presented in this study can be found in online repositories. The mass spectrometry proteomics data have been deposited to the ProteomeXchange Consortium via the PRIDE [1] partner repository with the dataset identifier PXD037909.
