# Supplementary material for: Energy metabolism in skeletal muscle cells from donors with different body mass index
Source: Front Physiol. 2022 Nov 17;13:982842. doi: 10.3389/fphys.2022.982842 (PMC9714574; doi:10.3389/fphys.2022.982842)
Supplement: Supplementary file 1 [file Table1.DOCX]

**Supplementary table 1:** Significantly upregulated proteins in myotubes from donors with obesity as compared to myotubes from lean donors (n=8 samples in each group)

| Gene | Protein | Fold change | Log p value |
| --- | --- | --- | --- |
| LIMCH1 | LIM and calponin homology domains-containing protein 1 | 4,35 | 1,69 |
| ACTBL2 | Beta-actin-like protein 2 | 3,42 | 1,31 |
| FAM188A | Protein FAM188A | 3,39 | 2,55 |
| CSNK2B | Casein kinase II subunit beta | 3,09 | 1,46 |
| CD36 | Platelet glycoprotein 4 | 3,01 | 1,78 |
| PVR | Poliovirus receptor | 2,95 | 2,04 |
| PPIL1 | Peptidyl-prolyl cis-trans isomerase-like 1 | 2,82 | 1,77 |
| MT1E | Metallothionein-1E | 2,73 | 1,71 |
| TNS3 | Tensin-3 | 2,62 | 1,58 |
| PRNP | Major prion protein | 2,60 | 1,74 |
| ENAM | Enamelin | 2,58 | 1,65 |
| TNFAIP8L3 | Tumor necrosis factor alpha-induced protein 8-like protein 3 | 2,56 | 1,72 |
| GIPC1 | PDZ domain-containing protein GIPC1 | 2,46 | 2,54 |
| HN1 | Hematological and neurological expressed 1 protein | 2,39 | 1,48 |
| LMO7 | LIM domain only protein 7 | 2,28 | 1,72 |
| RECK | Reversion-inducing cysteine-rich protein with Kazal motifs | 2,15 | 1,85 |
| CCND1 | G1/S-specific cyclin-D1 | 2,13 | 2,62 |
| CKB | Creatine kinase B-type | 2,10 | 1,57 |
| CTGF | Connective tissue growth factor | 2,01 | 1,70 |
| NEXN | Nexilin | 2,00 | 1,50 |
| SLC9A3R1 | Na(+)/H(+) exchange regulatory cofactor NHE-RF1 | 2,00 | 1,65 |
| PEA15 | Astrocytic phosphoprotein PEA-15 | 1,98 | 1,76 |
| RDH10 | Retinol dehydrogenase 10 | 1,93 | 1,72 |
| SERINC3 | Serine incorporator 3 | 1,93 | 1,37 |
| CRYAB | Alpha-crystallin B chain | 1,92 | 1,96 |
| DHRS3 | Short-chain dehydrogenase/reductase 3 | 1,92 | 1,51 |
| GLS | Glutaminase kidney isoform, mitochondrial | 1,91 | 1,70 |
| STRN4 | Striatin-4 | 1,90 | 1,64 |
| TMOD2 | Tropomodulin-2 | 1,89 | 1,43 |
| PDLIM4 | PDZ and LIM domain protein 4 | 1,88 | 1,78 |
| CDA | Cytidine deaminase | 1,87 | 1,39 |
| AHR | Aryl hydrocarbon receptor | 1,86 | 1,61 |
| SDPR | Serum deprivation-response protein | 1,86 | 1,74 |
| MRPS21 | 28S ribosomal protein S21, mitochondrial | 1,84 | 1,43 |
| PLCG1 | 1-phosphatidylinositol 4,5-bisphosphate phosphodiesterase gamma-1 | 1,83 | 1,43 |
| TRPV2 | Transient receptor potential cation channel subfamily V member 2 | 1,83 | 1,52 |
| LCMT1 | Leucine carboxyl methyltransferase 1 | 1,83 | 2,21 |
| C18orf8 | Uncharacterized protein C18orf8 | 1,83 | 1,72 |
| LGMN | Legumain | 1,76 | 1,30 |
| YPEL5 | Protein yippee-like 5 | 1,76 | 1,36 |
| PRPS1 | Ribose-phosphate pyrophosphokinase 1 | 1,74 | 1,71 |
| COMP | Cartilage oligomeric matrix protein | 1,62 | 1,37 |
| UFD1L | Ubiquitin fusion degradation protein 1 homolog | 1,62 | 2,02 |
| DYNLT3 | Dynein light chain Tctex-type 3 | 1,60 | 1,35 |
| BPGM | Bisphosphoglycerate mutase | 1,56 | 1,46 |
| TBC1D10B | TBC1 domain family member 10B | 1,56 | 2,04 |
| RTN4 | Reticulon-4 | 1,56 | 1,78 |
| SPECC1L | Cytospin-A | 1,56 | 1,71 |
| SLC25A4 | ADP/ATP translocase 1 | 1,54 | 1,51 |
| TMEM205 | Transmembrane protein 205 | 1,53 | 1,79 |
| PNP | Purine nucleoside phosphorylase | 1,53 | 1,40 |
| CNN2 | Calponin-2 | 1,53 | 1,40 |
| DNAJC5 | DnaJ homolog subfamily C member 5 | 1,53 | 1,40 |
| FHL2 | Four and a half LIM domains protein 2 | 1,50 | 2,54 |
| PDLIM7 | PDZ and LIM domain protein 7 | 1,50 | 1,60 |
| TRIM32 | E3 ubiquitin-protein ligase TRIM32 | 1,49 | 1,30 |
| TUBB8 | Tubulin beta-8 chain | 1,46 | 1,55 |
| HSPB6 | Heat shock protein beta-6 | 1,45 | 1,90 |
| HSPB1 | Heat shock protein beta-1 | 1,45 | 1,97 |
| DSTN | Destrin | 1,45 | 1,77 |
| MPRIP | Myosin phosphatase Rho-interacting protein | 1,45 | 1,73 |
| REEP5 | Receptor expression-enhancing protein 5 | 1,44 | 1,45 |
| FBXL18 | F-box/LRR-repeat protein 18 | 1,43 | 1,50 |
| CTPS1 | CTP synthase 1 | 1,43 | 1,62 |
| PPP1R12A | Protein phosphatase 1 regulatory subunit 12A | 1,43 | 1,61 |
| GPX1 | Glutathione peroxidase 1 | 1,42 | 1,31 |
| GSN | Gelsolin | 1,42 | 1,86 |
| ATP6V0A1 | V-type proton ATPase 116 kDa subunit a isoform 1 | 1,42 | 1,57 |
| MAN2B2 | Epididymis-specific alpha-mannosidase | 1,41 | 1,91 |
| DPP9 | Dipeptidyl peptidase 9 | 1,41 | 1,69 |
| ATOX1 | Copper transport protein ATOX1 | 1,40 | 1,74 |
| TOR1AIP2 | Torsin-1A-interacting protein 2 | 1,39 | 1,75 |
| SEC22B | Vesicle-trafficking protein SEC22b | 1,39 | 1,88 |
| MRPS36 | 28S ribosomal protein S36, mitochondrial | 1,39 | 2,51 |
| RRM2B | Ribonucleoside-diphosphate reductase subunit M2 B | 1,39 | 1,87 |
| ZYX | Zyxin | 1,39 | 1,70 |
| AHCY | Adenosylhomocysteinase | 1,37 | 2,02 |
| CRYL1 | Lambda-crystallin homolog | 1,37 | 1,36 |
| CLPTM1L | Cleft lip and palate transmembrane protein 1-like protein | 1,35 | 1,38 |
| DBN1 | Drebrin | 1,35 | 1,52 |
| MTCH1 | Mitochondrial carrier homolog 1 | 1,35 | 2,06 |
| YAP1 | Transcriptional coactivator YAP1 | 1,35 | 1,77 |
| DOHH | Deoxyhypusine hydroxylase | 1,34 | 1,76 |
| ARPC1A | Actin-related protein 2/3 complex subunit 1A | 1,34 | 2,41 |
| WDR26 | WD repeat-containing protein 26 | 1,34 | 1,82 |
| TXNDC17 | Thioredoxin domain-containing protein 17 | 1,34 | 1,68 |
| FKBP4 | Peptidyl-prolyl cis-trans isomerase FKBP4 | 1,33 | 1,68 |
| TIGAR | Fructose-2,6-bisphosphatase TIGAR | 1,33 | 1,36 |
| TCEB1 | Transcription elongation factor B polypeptide 1 | 1,33 | 1,55 |
| TXNRD1 | Thioredoxin reductase 1, cytoplasmic | 1,32 | 1,88 |
| IMPA1 | Inositol monophosphatase 1 | 1,32 | 1,41 |
| BCL2L13 | Bcl-2-like protein 13 | 1,32 | 2,14 |
| STUB1 | E3 ubiquitin-protein ligase CHIP | 1,31 | 1,65 |
| NUTF2 | Nuclear transport factor 2 | 1,31 | 1,66 |
| CACNA2D1 | Voltage-dependent calcium channel subunit alpha-2/delta-1 | 1,31 | 2,12 |
| MAP1B | Microtubule-associated protein 1B | 1,31 | 1,87 |
| TUBB6 | Tubulin beta-6 chain | 1,30 | 1,38 |
| PYGL | Glycogen phosphorylase, liver form | 1,30 | 1,56 |
| TUBB4B | Tubulin beta-4B chain | 1,29 | 1,58 |
| CLTA | Clathrin light chain A | 1,28 | 1,88 |
| CTTN | Src substrate cortactin | 1,28 | 1,73 |
| RFTN1 | Raftlin | 1,28 | 1,86 |
| GLO1 | Lactoylglutathione lyase | 1,28 | 2,14 |
| PCMT1 | Protein-L-isoaspartate(D-aspartate) O-methyltransferase | 1,27 | 3,75 |
| SRXN1 | Sulfiredoxin-1 | 1,27 | 1,53 |
| DUSP3 | Dual specificity protein phosphatase 3 | 1,27 | 1,48 |
| MYH9 | Myosin-9 | 1,27 | 1,35 |
| ACOT7 | Cytosolic acyl coenzyme A thioester hydrolase | 1,27 | 1,30 |
| PACS1 | Phosphofurin acidic cluster sorting protein 1 | 1,27 | 1,38 |
| PIP5K1A | Phosphatidylinositol 4-phosphate 5-kinase type-1 alpha | 1,26 | 1,48 |
| IPO7 | Importin-7 | 1,26 | 1,63 |
| PAFAH1B2 | Platelet-activating factor acetylhydrolase IB subunit beta | 1,25 | 1,41 |
| PPP6R1 | Serine/threonine-protein phosphatase 6 regulatory subunit 1 | 1,25 | 1,35 |
| HSPBP1 | Hsp70-binding protein 1 | 1,25 | 2,98 |
| TP53I3 | Quinone oxidoreductase PIG3 | 1,25 | 1,58 |
| VKORC1L1 | Vitamin K epoxide reductase complex subunit 1-like protein 1 | 1,23 | 2,46 |
| EXOC2 | Exocyst complex component 2 | 1,23 | 1,35 |
| PEBP1 | Phosphatidylethanolamine-binding protein 1 | 1,23 | 2,07 |
| TUBA1B | Tubulin alpha-1B chain | 1,23 | 1,35 |
| APOA1BP | NAD(P)H-hydrate epimerase | 1,22 | 2,24 |
| VTA1 | Vacuolar protein sorting-associated protein VTA1 homolog | 1,22 | 2,01 |
| CHMP1A | Charged multivesicular body protein 1a | 1,21 | 1,43 |
| AGL | Glycogen debranching enzyme | 1,21 | 1,78 |
| OPA1 | Dynamin-like 120 kDa protein, mitochondrial | 1,21 | 2,13 |
| HGS | Hepatocyte growth factor-regulated tyrosine kinase substrate | 1,21 | 1,81 |
| MCTS1 | Malignant T-cell-amplified sequence 1 | 1,21 | 2,44 |
| CAP1 | Adenylyl cyclase-associated protein 1 | 1,21 | 1,66 |
| EPB41L2 | Band 4.1-like protein 2 | 1,21 | 1,57 |
| FLAD1 | FAD synthase;Molybdenum cofactor biosynthesis protein-like region | 1,20 | 1,41 |
| PPP6R2 | Serine/threonine-protein phosphatase 6 regulatory subunit 2 | 1,20 | 1,44 |
| MAPK1 | Mitogen-activated protein kinase 1 | 1,20 | 1,43 |
| ST13 | Hsc70-interacting protein | 1,19 | 2,63 |
| HEBP2 | Heme-binding protein 2 | 1,19 | 2,14 |
| CORO1C | Coronin-1C | 1,19 | 1,45 |
| G3BP1 | Ras GTPase-activating protein-binding protein 1 | 1,18 | 1,34 |
| PPP1CA | Serine/threonine-protein phosphatase PP1-alpha catalytic subunit | 1,18 | 1,96 |
| UBE2K | Ubiquitin-conjugating enzyme E2 K | 1,18 | 1,87 |
| STIP1 | Stress-induced-phosphoprotein 1 | 1,18 | 1,48 |
| PRKACB | cAMP-dependent protein kinase catalytic subunit beta | 1,18 | 2,43 |
| CFL1 | Cofilin-1 | 1,17 | 1,83 |
| S100A16 | Protein S100-A16 | 1,16 | 1,57 |
| LANCL1 | LanC-like protein 1 | 1,16 | 1,43 |
| TUBB | Tubulin beta chain | 1,16 | 1,56 |
| PSMA5 | Proteasome subunit alpha type-5 | 1,16 | 1,45 |
| COPS5 | COP9 signalosome complex subunit 5 | 1,16 | 1,76 |
| UBE2N | Ubiquitin-conjugating enzyme E2 N | 1,16 | 2,31 |
| USP14 | Ubiquitin carboxyl-terminal hydrolase 14 | 1,16 | 2,36 |
| PSMD8 | 26S proteasome non-ATPase regulatory subunit 8 | 1,15 | 1,33 |
| PTPN11 | Tyrosine-protein phosphatase non-receptor type 11 | 1,15 | 1,46 |
| CCT5 | T-complex protein 1 subunit epsilon | 1,15 | 2,41 |
| PFN1 | Profilin-1 | 1,15 | 1,33 |
| SERINC1 | Serine incorporator 1 | 1,15 | 1,64 |
| PSMB6 | Proteasome subunit beta type-6 | 1,15 | 1,68 |
| SBDS | Ribosome maturation protein SBDS | 1,15 | 1,44 |
| TUBA1C | Tubulin alpha-1C chain | 1,15 | 1,33 |
| TXNL1 | Thioredoxin-like protein 1 | 1,14 | 1,59 |
| PFDN5 | Prefoldin subunit 5 | 1,14 | 1,94 |
| PGK1 | Phosphoglycerate kinase 1 | 1,13 | 1,43 |
| CCT4 | T-complex protein 1 subunit delta | 1,13 | 2,13 |
| CCT3 | T-complex protein 1 subunit gamma | 1,13 | 1,61 |
| DYNLRB1 | Dynein light chain roadblock-type 1 | 1,13 | 1,80 |
| LDHB | L-lactate dehydrogenase B chain | 1,12 | 1,34 |
| OLA1 | Obg-like ATPase 1 | 1,12 | 1,37 |
| ACTR3 | Actin-related protein 3 | 1,12 | 1,66 |
| PRKAR1A | cAMP-dependent protein kinase type I-alpha regulatory subunit | 1,12 | 1,44 |
| PFDN2 | Prefoldin subunit 2 | 1,12 | 1,49 |
| CCT7 | T-complex protein 1 subunit eta | 1,11 | 1,87 |
| TCP1 | T-complex protein 1 subunit alpha | 1,11 | 1,47 |
| RTCB | tRNA-splicing ligase RtcB homolog | 1,11 | 1,63 |
| COPS6 | COP9 signalosome complex subunit 6 | 1,09 | 1,49 |
| PSMC3 | 26S protease regulatory subunit 6A | 1,08 | 1,47 |
| EIF3F | Eukaryotic translation initiation factor 3 subunit F | 1,07 | 1,48 |
