# Supplementary material for: Energy metabolism in skeletal muscle cells from donors with different body mass index
Source: Front Physiol. 2022 Nov 17;13:982842. doi: 10.3389/fphys.2022.982842 (PMC9714574; doi:10.3389/fphys.2022.982842)
Supplement: Supplementary file 2 [file Table2.DOCX]

**Supplementary table 2:** Significantly downregulated proteins in myotubes from donors with obesity as compared to myotubes from lean donors (n=8 samples in each group)

| Gene | Protein | Fold change | Log p value |
| --- | --- | --- | --- |
| PTMA | Prothymosin alpha | -7,13 | 1,35 |
| CHI3L1 | Chitinase-3-like protein 1 | -6,64 | 2,05 |
| C3 | Complement C3 | -5,63 | 1,59 |
| PPL | Periplakin | -5,50 | 2,10 |
| ALPL | Alkaline phosphatase, tissue-nonspecific isozyme | -3,57 | 1,67 |
| MPG | DNA-3-methyladenine glycosylase | -3,55 | 1,70 |
| CLU | Clusterin;Clusterin beta chain;Clusterin alpha chain | -3,33 | 2,09 |
| BABAM1 | BRISC and BRCA1-A complex member 1 | -3,21 | 2,14 |
| PLIN2 | Perilipin-2 | -3,04 | 2,16 |
| RGPD3 | RanBP2-like and GRIP domain-containing protein 3 | -2,68 | 2,64 |
| TREX1 | Three-prime repair exonuclease 1 | -2,57 | 1,77 |
| SLC27A3 | Long-chain fatty acid transport protein 3 | -2,53 | 1,39 |
| SLC16A7 | Monocarboxylate transporter 2 | -2,49 | 2,34 |
| SULT1A4 | Sulfotransferase 1A4;Sulfotransferase 1A3 | -2,48 | 2,07 |
| HMGCS1 | Hydroxymethylglutaryl-CoA synthase, cytoplasmic | -2,44 | 1,41 |
| TMEM189 | Transmembrane protein 189 | -2,41 | 1,46 |
| AIM1 | Absent in melanoma 1 protein | -2,37 | 1,71 |
| SAP18 | Histone deacetylase complex subunit SAP18 | -2,35 | 1,35 |
| MAGOHB | Protein mago nashi homolog 2 | -2,34 | 2,01 |
| CYP51A1 | Lanosterol 14-alpha demethylase | -2,30 | 3,44 |
| MRPS11 | 28S ribosomal protein S11, mitochondrial | -2,26 | 1,47 |
| COL14A1 | Collagen alpha-1(XIV) chain | -2,25 | 1,35 |
| C1S | Complement C1s subcomponent | -2,24 | 1,51 |
| HSD11B1 | Corticosteroid 11-beta-dehydrogenase isozyme 1 | -2,22 | 1,38 |
| CTSL | Cathepsin L1 | -2,18 | 2,33 |
| ERAP1 | Endoplasmic reticulum aminopeptidase 1 | -2,11 | 3,80 |
| C14orf159 | UPF0317 protein C14orf159, mitochondrial | -2,09 | 1,57 |
| TRIM47 | Tripartite motif-containing protein 47 | -2,09 | 1,55 |
| CNTNAP1 | Contactin-associated protein 1 | -2,06 | 2,04 |
| DOCK11 | Dedicator of cytokinesis protein 11 | -2,06 | 1,62 |
| SMCHD1 | Structural maintenance of chromosomes flexible hinge  domain-containing protein 1 | -2,05 | 1,60 |
| GBP2 | Interferon-induced guanylate-binding protein 2 | -2,03 | 1,99 |
| PARP9 | Poly [ADP-ribose] polymerase 9 | -2,02 | 1,53 |
| CA12 | Carbonic anhydrase 12 | -2,02 | 1,98 |
| NADSYN1 | Glutamine-dependent NAD(+) synthetase | -2,00 | 1,77 |
| STRIP1 | Striatin-interacting protein 1 | -1,97 | 1,52 |
| EXOSC5 | Exosome complex component RRP46 | -1,97 | 2,02 |
| PC | Pyruvate carboxylase, mitochondrial | -1,96 | 1,33 |
| TSPAN14 | Tetraspanin-14 | -1,94 | 1,32 |
| PSMB9 | Proteasome subunit beta type-9 | -1,91 | 1,66 |
| ABHD2 | Abhydrolase domain-containing protein 2 | -1,89 | 1,69 |
| TYMP | Thymidine phosphorylase | -1,88 | 1,50 |
| LAMA4 | Laminin subunit alpha-4 | -1,88 | 1,67 |
| GLUL | Glutamine synthetase | -1,87 | 1,37 |
| MRGPRF | Mas-related G-protein coupled receptor member F | -1,86 | 1,53 |
| ARHGAP6 | Rho GTPase-activating protein 6 | -1,86 | 1,61 |
| MSH2 | DNA mismatch repair protein Msh2 | -1,86 | 1,70 |
| AMDHD2 | Putative N-acetylglucosamine-6-phosphate deacetylase | -1,85 | 1,39 |
| SKIV2L2 | Superkiller viralicidic activity 2-like 2 | -1,85 | 1,65 |
| AARS2 | Alanine--tRNA ligase, mitochondrial | -1,84 | 1,49 |
| UBA7 | Ubiquitin-like modifier-activating enzyme 7 | -1,77 | 2,25 |
| LDLR | Low-density lipoprotein receptor | -1,77 | 1,39 |
| PTPRG | Receptor-type tyrosine-protein phosphatase gamma | -1,77 | 2,41 |
| NUCKS1 | Nuclear ubiquitous casein and cyclin-dependent kinase substrate 1 | -1,76 | 1,83 |
| SRSF6 | Serine/arginine-rich splicing factor 6 | -1,75 | 1,55 |
| CERCAM | Probable inactive glycosyltransferase 25 family member 3 | -1,75 | 2,27 |
| SLC39A14 | Zinc transporter ZIP14 | -1,71 | 2,13 |
| SELENBP1 | Selenium-binding protein 1 | -1,70 | 1,63 |
| CBR3 | Carbonyl reductase [NADPH] 3 | -1,68 | 1,31 |
| PSEN1 | Presenilin-1 | -1,68 | 1,73 |
| LACC1 | Laccase domain-containing protein 1 | -1,68 | 2,00 |
| GFPT2 | Glutamine--fructose-6-phosphate aminotransferase [isomerizing] 2 | -1,65 | 1,97 |
| UTP6 | U3 small nucleolar RNA-associated protein 6 homolog | -1,64 | 1,55 |
| LEPREL2 | Prolyl 3-hydroxylase 3 | -1,64 | 1,55 |
| OPLAH | 5-oxoprolinase | -1,63 | 1,74 |
| TBC1D2B | TBC1 domain family member 2B | -1,63 | 1,58 |
| WDR82 | WD repeat-containing protein 82 | -1,61 | 1,85 |
| EBP | 3-beta-hydroxysteroid-Delta(8),Delta(7)-isomerase | -1,60 | 1,87 |
| SUN1 | SUN domain-containing protein 1 | -1,60 | 1,38 |
| SAMD9 | Sterile alpha motif domain-containing protein 9 | -1,60 | 1,42 |
| SUN2 | SUN domain-containing protein 2 | -1,60 | 1,51 |
| CNPY3 | Protein canopy homolog 3 | -1,59 | 3,49 |
| SPRYD4 | SPRY domain-containing protein 4 | -1,58 | 1,51 |
| COL5A2 | Collagen alpha-2(V) chain | -1,55 | 1,44 |
| TKT | Transketolase | -1,55 | 1,93 |
| PNO1 | RNA-binding protein PNO1 | -1,55 | 1,97 |
| PLEKHF1 | Pleckstrin homology domain-containing family F member 1 | -1,53 | 1,53 |
| SDCBP | Syntenin-1 | -1,52 | 1,34 |
| UBTF | Nucleolar transcription factor 1 | -1,50 | 1,55 |
| UBE2L6 | Ubiquitin/ISG15-conjugating enzyme E2 L6 | -1,48 | 1,40 |
| ANP32B | Acidic leucine-rich nuclear phosphoprotein 32 family member B | -1,48 | 1,41 |
| NR3C1 | Glucocorticoid receptor | -1,47 | 1,39 |
| ANP32A | Acidic leucine-rich nuclear phosphoprotein 32 family member A | -1,46 | 1,48 |
| LAMC1 | Laminin subunit gamma-1 | -1,46 | 1,34 |
| SRSF10 | Serine/arginine-rich splicing factor 10 | -1,45 | 1,46 |
| H6PD | Glucose 1-dehydrogenase | -1,44 | 1,53 |
| MYCBP2 | E3 ubiquitin-protein ligase MYCBP2 | -1,44 | 1,36 |
| DNAJC10 | DnaJ homolog subfamily C member 10 | -1,44 | 2,51 |
| ITM2B | Integral membrane protein 2B | -1,43 | 1,49 |
| FBN1 | Fibrillin-1 | -1,43 | 1,32 |
| FNDC3A | Fibronectin type-III domain-containing protein 3A | -1,42 | 1,35 |
| MRPL13 | 39S ribosomal protein L13, mitochondrial | -1,42 | 1,52 |
| DAK | Bifunctional ATP-dependent dihydroxyacetone kinase/FAD-AMP lyase | -1,41 | 2,96 |
| PLSCR1 | Phospholipid scramblase 1 | -1,41 | 1,31 |
| CISD1 | CDGSH iron-sulfur domain-containing protein 1 | -1,41 | 2,07 |
| WIBG | Partner of Y14 and mago | -1,40 | 1,39 |
| SLC30A1 | Zinc transporter 1 | -1,40 | 1,46 |
| SAFB | Scaffold attachment factor B1 | -1,40 | 1,58 |
| RAVER1 | Ribonucleoprotein PTB-binding 1 | -1,40 | 1,37 |
| ASL | Argininosuccinate lyase | -1,39 | 1,50 |
| RNF213 | E3 ubiquitin-protein ligase RNF213 | -1,39 | 1,48 |
| NOL6 | Nucleolar protein 6 | -1,39 | 1,78 |
| PNMA2 | Paraneoplastic antigen Ma2 | -1,39 | 2,89 |
| KPNA2 | Importin subunit alpha-1 | -1,38 | 1,47 |
| CPSF1 | Cleavage and polyadenylation specificity factor subunit 1 | -1,38 | 1,42 |
| PIR | Pirin | -1,38 | 1,91 |
| GPAA1 | Glycosylphosphatidylinositol anchor attachment 1 protein | -1,38 | 1,62 |
| LEPRE1 | Prolyl 3-hydroxylase 1 | -1,36 | 1,64 |
| DOLPP1 | Dolichyldiphosphatase 1 | -1,36 | 1,54 |
| AKR1C3 | Aldo-keto reductase family 1 member C3 | -1,35 | 1,46 |
| ELP2 | Elongator complex protein 2 | -1,35 | 1,36 |
| TM9SF4 | Transmembrane 9 superfamily member 4 | -1,35 | 2,69 |
| POGLUT1 | Protein O-glucosyltransferase 1 | -1,34 | 1,78 |
| MRPL9 | 39S ribosomal protein L9, mitochondrial | -1,33 | 1,71 |
| RPRD1B | Regulation of nuclear pre-mRNA domain-containing protein 1B | -1,33 | 1,69 |
| ASPH | Aspartyl/asparaginyl beta-hydroxylase | -1,33 | 1,94 |
| RAE1 | mRNA export factor | -1,33 | 1,53 |
| CRTAP | Cartilage-associated protein | -1,32 | 1,83 |
| TMEM43 | Transmembrane protein 43 | -1,31 | 1,93 |
| LMNA | Prelamin-A/C;Lamin-A/C | -1,31 | 1,42 |
| NNMT | Nicotinamide N-methyltransferase | -1,31 | 2,16 |
| VAC14 | Protein VAC14 homolog | -1,30 | 1,44 |
| GALK1 | Galactokinase | -1,30 | 2,69 |
| RPL28 | 60S ribosomal protein L28 | -1,30 | 1,42 |
| PDIA6 | Protein disulfide-isomerase A6 | -1,30 | 1,93 |
| PCNA | Proliferating cell nuclear antigen | -1,29 | 1,40 |
| CNPY2 | Protein canopy homolog 2 | -1,29 | 1,69 |
| SYMPK | Symplekin | -1,29 | 1,33 |
| PLOD1 | Procollagen-lysine,2-oxoglutarate 5-dioxygenase 1 | -1,29 | 2,62 |
| SLC30A7 | Zinc transporter 7 | -1,29 | 1,44 |
| SERBP1 | Plasminogen activator inhibitor 1 RNA-binding protein | -1,29 | 1,50 |
| CNBP | Cellular nucleic acid-binding protein | -1,28 | 1,74 |
| STAT2 | Signal transducer and activator of transcription 2 | -1,28 | 2,14 |
| MRPL49 | 39S ribosomal protein L49, mitochondrial | -1,27 | 1,54 |
| TOM1 | Target of Myb protein 1 | -1,27 | 2,23 |
| RAB34 | Ras-related protein Rab-34 | -1,27 | 1,76 |
| IGF2R | Cation-independent mannose-6-phosphate receptor | -1,27 | 1,33 |
| DHRS4 | Dehydrogenase/reductase SDR family member 4 | -1,27 | 1,40 |
| ERH | Enhancer of rudimentary homolog | -1,26 | 1,40 |
| PHPT1 | 14 kDa phosphohistidine phosphatase | -1,26 | 1,48 |
| NME3 | Nucleoside diphosphate kinase 3 | -1,25 | 1,70 |
| HINT2 | Histidine triad nucleotide-binding protein 2, mitochondrial | -1,25 | 1,64 |
| ZC3H15 | Zinc finger CCCH domain-containing protein 15 | -1,25 | 2,02 |
| PGD | 6-phosphogluconate dehydrogenase, decarboxylating | -1,25 | 1,41 |
| DHX15 | Pre-mRNA-splicing factor ATP-dependent RNA helicase DHX15 | -1,25 | 1,41 |
| COX4I1 | Cytochrome c oxidase subunit 4 isoform 1, mitochondrial | -1,25 | 1,51 |
| TBC1D15 | TBC1 domain family member 15 | -1,24 | 1,61 |
| CAD | Glutamine-dependent carbamoyl-phosphate synthase | -1,24 | 1,46 |
| FHOD1 | FH1/FH2 domain-containing protein 1 | -1,24 | 1,31 |
| TMTC3 | Transmembrane and TPR repeat-containing protein 3 | -1,23 | 1,48 |
| PDIA4 | Protein disulfide-isomerase A4 | -1,23 | 2,01 |
| COLGALT1 | Procollagen galactosyltransferase 1 | -1,23 | 1,49 |
| SF3B3 | Splicing factor 3B subunit 3 | -1,23 | 1,37 |
| NDUFA5 | NADH dehydrogenase [ubiquinone] 1 alpha subcomplex subunit 5 | -1,22 | 1,44 |
| GMPPA | Mannose-1-phosphate guanyltransferase alpha | -1,22 | 1,37 |
| G6PD | Glucose-6-phosphate 1-dehydrogenase | -1,22 | 1,71 |
| PIGS | GPI transamidase component PIG-S | -1,21 | 2,09 |
| IDH1 | Isocitrate dehydrogenase [NADP] cytoplasmic | -1,21 | 1,67 |
| HSPA1B | Heat shock 70 kDa protein 1B;Heat shock 70 kDa protein 1A | -1,21 | 1,35 |
| RRBP1 | Ribosome-binding protein 1 | -1,21 | 3,35 |
| PTPN23 | Tyrosine-protein phosphatase non-receptor type 23 | -1,20 | 1,35 |
| HYOU1 | Hypoxia up-regulated protein 1 | -1,20 | 1,92 |
| SRPRB | Signal recognition particle receptor subunit beta | -1,20 | 2,00 |
| SEC61A1 | Protein transport protein Sec61 subunit alpha isoform 1 | -1,20 | 1,56 |
| PCYT1A | Choline-phosphate cytidylyltransferase A | -1,20 | 1,64 |
| ARMC10 | Armadillo repeat-containing protein 10 | -1,20 | 1,97 |
| HSP90B1 | Endoplasmin | -1,20 | 2,53 |
| ERP29 | Endoplasmic reticulum resident protein 29 | -1,20 | 1,31 |
| RPL38 | 60S ribosomal protein L38 | -1,20 | 1,47 |
| CALR | Calreticulin | -1,19 | 2,33 |
| ALDH9A1 | 4-trimethylaminobutyraldehyde dehydrogenase | -1,19 | 1,33 |
| UGGT1 | UDP-glucose:glycoprotein glucosyltransferase 1 | -1,19 | 1,50 |
| NDUFB10 | NADH dehydrogenase [ubiquinone] 1 beta subcomplex subunit 10 | -1,19 | 1,42 |
| RPS15A | 40S ribosomal protein S15a | -1,19 | 1,70 |
| SRP72 | Signal recognition particle subunit SRP72 | -1,18 | 1,86 |
| DDOST | Dolichyl-diphosphooligosaccharide-protein glycosyltransferase | -1,18 | 3,21 |
| EDF1 | Endothelial differentiation-related factor 1 | -1,18 | 1,41 |
| TBL2 | Transducin beta-like protein 2 | -1,18 | 1,37 |
| AGPS | Alkyldihydroxyacetonephosphate synthase, peroxisomal | -1,17 | 1,82 |
| ZC3HAV1 | Zinc finger CCCH-type antiviral protein 1 | -1,16 | 1,81 |
| RPN2 | Dolichyl-diphosphooligosaccharide-protein glycosyltransferase subunit 2 | -1,16 | 1,67 |
| CNOT1 | CCR4-NOT transcription complex subunit 1 | -1,16 | 1,66 |
| HSPA5 | 78 kDa glucose-regulated protein | -1,16 | 2,77 |
| GMPPB | Mannose-1-phosphate guanyltransferase beta | -1,16 | 2,30 |
| CACYBP | Calcyclin-binding protein | -1,16 | 1,67 |
| RPS3A | 40S ribosomal protein S3a | -1,14 | 1,64 |
| RPS11 | 40S ribosomal protein S11 | -1,14 | 2,76 |
| TALDO1 | Transaldolase | -1,14 | 1,71 |
| POR | NADPH--cytochrome P450 reductase | -1,14 | 1,31 |
| PSME2 | Proteasome activator complex subunit 2 | -1,13 | 1,60 |
| PRDX5 | Peroxiredoxin-5, mitochondrial | -1,12 | 1,52 |
| ARCN1 | Coatomer subunit delta | -1,12 | 1,74 |
| PDAP1 | 28 kDa heat- and acid-stable phosphoprotein | -1,12 | 1,39 |
| RDH11 | Retinol dehydrogenase 11 | -1,11 | 2,24 |
| RPLP0 | 60S acidic ribosomal protein P0;60S acidic ribosomal protein P0-like | -1,11 | 1,37 |
| RPN1 | Dolichyl-diphosphooligosaccharide protein glycosyltransferase subunit 1 | -1,11 | 1,30 |
| ECM29 | Proteasome-associated protein ECM29 homolog | -1,10 | 1,47 |
| EIF3I | Eukaryotic translation initiation factor 3 subunit I | -1,08 | 1,41 |
| SEC23A | Protein transport protein Sec23A | -1,06 | 1,39 |
